# THE DIFFERENTIAL INFLUENCE OF REGIONAL CONTEXT ON LATER-LIFE HEALTH AND MORTALITY

**DOI:** 10.1093/geroni/igz038.2970

**Published:** 2019-11-08

**Authors:** Alicia Riley

**Affiliations:** University of Chicago, Chicago, Illinois, United States

## Abstract

This study examines regional disparities in later life health from a life course perspective. To sort out when and how region influences health over the life course, I focus on the sharp contrast between the South and the rest of the U.S. in health and mortality. I draw on data from the National Life Health and Aging Project (NSHAP), a nationally representative sample of community-dwelling older adults in the U.S., to estimate the differential risk of multiple health outcomes and mortality by regional trajectory. I find that older adults who leave the South are worse off in multiple outcomes than those who stay. I also find evidence of a protective health effect of community cohesion and dense social networks for the Southerners who stay in the South. My results suggest that regional trajectory influences health in later life through its associations with socioeconomic status, access to healthcare, and social rootedness.

